# Accurate determination of CT point‐spread‐function with high precision

**DOI:** 10.1120/jacmp.v14i4.3905

**Published:** 2013-07-08

**Authors:** Akihiro Kayugawa, Masaki Ohkubo, Shinichi Wada

**Affiliations:** ^1^ Niigata University Medical and Dental Hospital Niigata Japan; ^2^ Graduate School of Health Sciences Niigata University Niigata Japan

**Keywords:** computed tomography (CT), point spread function (PSF), modulation transfer function (MTF), line spread function (LSF), spatial resolution

## Abstract

The measurement of modulation transfer functions (MTFs) in computed tomography (CT) is often performed by scanning a point source phantom such as a thin wire or a microbead. In these methods the region of interest (ROI) is generally placed on the scanned image to crop the point source response. The aim of the present study was to examine the effect of ROI size on MTF measurement, and to optimize the ROI size. Using a 4 multidetector‐row CT, MTFs were measured by the wire and bead methods for three types of reconstruction kernels designated as ‘smooth', ‘standard', and ‘edge‐enhancement’ kernels. The size of a square ROI was changed from 30 to 50 pixels (approximately 2.9 to 4.9 mm). The accuracies of the MTFs were evaluated using the verification method. The MTFs measured by the wire and bead methods were dependent on ROI size, particularly in MTF measurement for the ‘edge‐enhancement’ kernel. MTF accuracy evaluated by the verification method changed with ROI size, and we were able to determine the optimum ROI size for each method (wire/bead) and for each kernel. Using these optimal ROI sizes, the MTF obtained by the wire method was in strong agreement with the MTF obtained by the bead method in each kernel. Our data demonstrate that the difficulties in obtaining accurate MTFs for some kernels such as edge‐enhancement can be overcome by incorporating the verification method into the wire and bead methods, allowing optimization of the ROI size to accurately determine the MTF.

PACS numbers: 87.57.‐s, 87.57.cf, 87.57.Q‐

## I. INTRODUCTION

To characterize the image sharpness of computed tomography (CT), point spread function (PSF) and modulation transfer function (MTF) are frequently employed. The PSF and MTF are used in basic studies evaluating the physical performance of a system,^1^, ^2^ and are also applied to image simulations of small structures^3^, ^4^ and to image filtering techniques.^5^, ^6^ In all these procedures, it is critical to determine PSF and MTF with high precision and accuracy.

**Figure 1:**
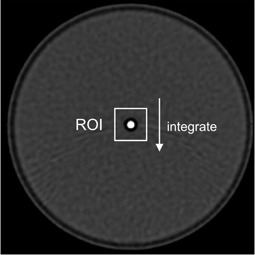
Schematic explanation of the data processing on the point source image for obtaining the LSF by integrating the ROI.

To evaluate the image sharpness in the scan plane, MTF measurements are performed by using a phantom such as point source,^7^, ^8^, ^9^ line pair,^(10)^ edge,^(11)^ as well as others.^(12)^ Methods that use a point source phantom, such as a thin wire or a microbead, are most widely utilized in current CT systems^13^, ^14^, ^15^, ^16^, ^17^, ^18^, ^19^, ^20^ as they are conceptual simplicity and relatively easy to implement. When using such methods, in a typical approach for determination of MTF,^7^, ^8^, ^9^, ^12^, ^13^, ^14^, ^15^, ^16^, ^17^, ^18^, ^19^ the line spread function (LSF) is first obtained by processing the scanned image (Fig. 1) in which the region of interest (ROI) placed on the point source response is integrated in one of the matrix directions. The MTF is then derived from the LSF using a Fourier transform. Despite its widespread use, a detailed methodology has only been examined for single‐slice CT.^21^, ^22^ In studies examining MTF measurement on early EMI CT,^(21)^ the size of the ROI used for the integration (Fig. 1) was suggested to affect the resultant MTF. The ROI size for obtaining accurate MTF values has also been extensively studied in a computed radiography system.^23^, ^24^ However, to our knowledge, there are no prior studies examining the optimum ROI size in a multidetector‐row CT (MDCT).

For the validation of MTF, the measured MTFs are generally compared with those obtained by other methods, or the reproducibility of measurements is determined.^10^, ^11^, ^25^ However, a more effective method to verify the measured MTF was reported,^(26)^ which enables the evaluation of MTF accuracy by comparing the measured image and the computed image based on the theory of image blurring described by the MTF. In the present study, we examined the MTF dependency on ROI size in the wire and bead methods, and applied the MTF verification method^(26)^ to evaluate the accuracy of MTFs obtained by the wire/bead methods. Using this approach we were able to optimize the ROI size and effectively evaluate image quality.

## II. MATERIALS AND METHODS

### A. MTF measurement methods

#### A.1 Wire method

**Figure 2:**
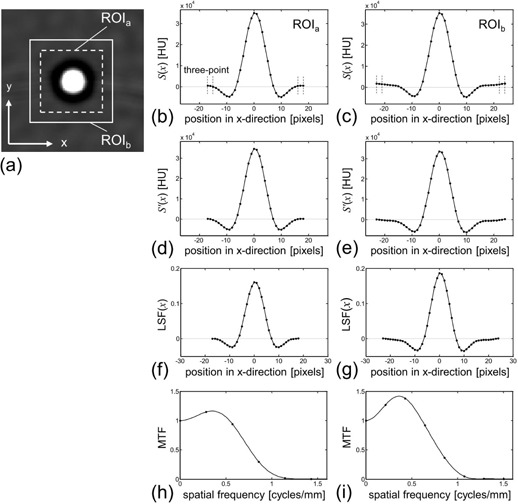
MTF measurement process. Wire phantom image (a) reconstructed with the kernel of FC52. Two square ROIs (${\text{ROI}}_{a}$ and ${\text{ROI}}_{b}$) were placed on the image, with ROI sizes of 36 pixels (${\text{ROI}}_{a}$) and 48 pixels (${\text{ROI}}_{b}$). The S(x) (b)–(c) obtained by Eq. (1) for ${\text{ROI}}_{a}$ and ${\text{ROI}}_{b}$, respectively. The $S^{{\;}^{\prime }}(x)$ (d)–(e) obtained by offset‐correction from the S(x) shown in (b) and (c), respectively. The LSF (f)–(g) obtained by normalization from the $S^{{\;}^{\prime }}(x)$ shown in (d) and (e), respectively. The MTF (h)–(i) obtained by zero‐padding and Fourier transform from the LSF shown in (f) and (g), respectively.

We determined the MTFs from the images obtained by scanning a thin wire. Each step in this process is shown in Fig. 2, where two ROIs (${\text{ROI}}_{a}$ and ${\text{ROI}}_{b}$) were used as examples. First, we obtained S(x) by integrating the ROI in y direction, as follows:

(1) $$S(x)=\sum_{y}\text{ROI}(x,y)$$

where *x* and *y* are the spatial locations of the pixel in the ROI, and ROI(x,y) is the pixel CT value (Hounsfield unit: HU) at the location of (x,y). The S(x) obtained for each ROI is shown in Figs. 2(b) and 2(c). Three‐point data values in both sides of the S(x) (six‐point data values in total) were averaged (Fig. 2), and we regarded the averaged value as the offset of the S(x) from 0 HU. The offset value for ${\text{ROI}}_{b}$ was higher than that for ${\text{ROI}}_{a}$, and the difference was induced by the difference of ROI sizes.

Next, we defined the $S^{{\;}^{\prime }}(x)$ that was obtained by subtracting the offset value from S(x) (Figs. 2(d) and 2(e)). As a result of this offset correction, the $S^{{\;}^{\prime }}(x)$ for ${\text{ROI}}_{b}$ had a larger negative wing than for ${\text{ROI}}_{a}$. The LSF was then obtained by normalizing the $S^{{\;}^{\prime }}(x)$, as follows:

(2) $$\text{LSF}(x)=S^{\prime }(x)/\sum_{x}S^{\prime }(x)$$

The LSF obtained for each ROI is shown in Figs. 2(f) and 2(g). As a result of this normalization, the LSF for ${\text{ROI}}_{b}$ had a larger range between the minimum and maximum values than the LSF for ${\text{ROI}}_{a}$ because of the large negative wing of $S^{{\;}^{\prime }}(x)$ for ${\text{ROI}}_{b}$.

Finally, the LSF was zero padded (to 512 points) and Fourier transformed, resulting in the determination of the MTF, as follows:

(3) MTF(w)=|F[zero padded LSF(x)]|

where *w* represents the spatial frequency and *F* denotes the Fourier transform. By the normalization in Eq. (2), the MTF had a value of 1.0 at the zero spatial frequency. The obtained MTF for each ROI is shown in Figs. 2(h) and 2(i).

As above, the process used for MTF determination in this study was thoroughly described, and we did not perform any other processing such as curve‐fitting/smoothing of our data. An example of the MTF dependency on the size of ROI for integration is shown in Fig. 2. We used a thin copper wire phantom with a 0.18 mm diameter,^(22)^ which was included in a cylinder of 50 mm diameter filled with water. The phantom was then scanned using the scan and reconstruction parameters described below. On the wire phantom image, we placed the square ROI of sizes differing from 30 to 50 pixels (approximately 2.9 to 4.9 mm) in 1 pixel increments (approximately 0.1 mm). Twenty‐one MTF results were obtained from one wire image.

#### A.2 Bead method

We also determined the MTFs using a microbead (0.28 mm diameter^(17)^). We utilized the Catphan 600 phantom (The Phantom Laboratory, Salem, NY) in which the microbead was included (CTP591 module).^(9)^ The scan and reconstruction parameters are described below. The process used for MTF determination from the image of the bead phantom was the same as for the wire method, and 21 MTF results were obtained from one bead image.

### B. Verification of measured MTF

To validate the MTFs measured by the wire and bead methods, we applied a previously proposed verification method^(26)^ with slight modifications. In brief, we assume that the CT image I(x,y) of an object‐function O(x,y) is characterized by the spatial resolution in the xy scan plane, in which the object‐function does not vary at any position in the Z direction perpendicular to the scan plane. We also assume that the imaging system is isotropic (i.e., the spatial resolution in the scan plane has rotational symmetry^3^, ^4^, ^6^). The image I(x,y) is then expressed as follows:^7^, ^8^, ^27^

(4) $$I(x,y)=F^{-1}\{F[O(x,y)].\text{MTF}(w)\}$$

(5) $$w=\sqrt{u^{2}+v^{2}}$$

where $F^{-1}$ denotes the inverse Fourier transform, *u* and *v* are the spatial frequency coordinates in the x and y directions, respectively, and *w* is spatial frequency in the radial direction. Noise and artifact components are neglected. By Eq. (4), the image for an assumed object‐function is calculated using the measured MTF. When the MTF was accurate, the calculated image agreed well to the scanned image of the phantom corresponding to the object‐function. The difference between the calculated and scanned images depends on the accuracy of the measured MTF. Then, by comparison of those images, the accuracy of MTF is estimated.

**Figure 3:**
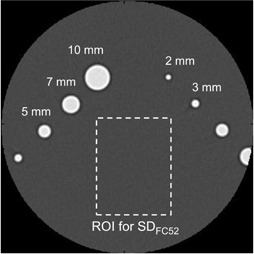
Scanned image of the high‐contrast CT test phantom. Cylindrical objects with diameters of 10, 7, 5, 3, and 2 mm were embedded in the phantom. The image noise was evaluated by the standard deviation (‘${\text{SD}}_{\text{FC}52}$’ for the image reconstructed with the kernel of FC52) in the ROI.

**Figure 4:**
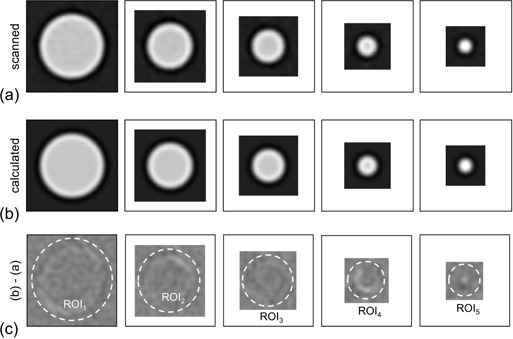
Images of the cylindrical objects with 10, 7, 5, 3, and 2 mm diameters. Scanned images (a) for the reconstruction kernel of FC52. Each image was obtained by cropping a rectangular region around each cylindrical object on the image in Fig. 3. Calculated images (b) by Eq. (5) using the measured MTF for the kernel FC52. Images (c) obtained by subtracting the scanned images (a) from the calculated images (b). The circular ROI (${\text{ROI}}_{1,2,\ldots 5}$) was placed on the image that had a radius of each objects radius plus 1.5 mm.

The phantom used in the verification method was a commercial phantom (high‐contrast CT test phantom (MHT‐type, Kyoto Kagaku Co. Ltd., Kyoto, Japan),^4^, ^6^, ^26^ which was filled with lung tissue‐equivalent material and included uniform cylindrical objects made of soft‐tissue–equivalent material. The image of this high‐contrast phantom is shown in Fig. 3, which was reconstructed with the kernel of FC52 (described in Materials & Methods Section C, below) as an example. The ROI shown in Fig. 3 was used to obtain the standard deviation (‘${\text{SD}}_{\text{FC}52}$', defined later in this section). The five cylindrical objects with diameters of 10, 7, 5, 3, and 2 mm in the phantom were used for verification. One example of the scanned image for each cylinder is shown in Fig. 4(a), in which images were obtained by a linear interpolation with the fine pixel size (0.04 mm) described below. The object‐function O(x,y) in Eq. (4) was numerically generated so that it simulated the shape and the CT value for each cylinder in the phantom as diameter‐known ideal cylinders. To avoid influence of the aliasing error, the O(x,y) was generated sufficiently fine in 0.04 mm pixel size. The measured MTF was resampled by a linear interpolation with the same data interval as the F[O(x,y)] in the spatial frequency domain. This allowed the numerical calculation in Eq. (4), and the image I(x,y) was obtained. An example of I(x,y) is shown in Fig. 4(b). These calculated images were compared with corresponding scanned images. To quantitatively evaluate the image differences, the SD of the subtraction image was used in the present study. The SD values were also used for evaluating the image noise level, as described later in this section. On five subtraction images (Fig. 4(c)), the SDs were obtained in the ROIs (${\text{ROI}}_{1,2,\ldots 5}$), termed ${\text{SD}}_{1,2,\ldots 5}$. We defined the ${\text{SD}}_{\text{average}}$ as follows:

(6) $${\text{SD}}_{\text{average}}=\frac{1}{5}\left(\sum_{i=1}^{5}{\text{SD}}_{i}\right)$$

We investigated the correlation between the ${\text{SD}}_{\text{average}}$ and the ROI size in the MTF measurements with wire/bead methods (Fig. 2).

The measured ${\text{SD}}_{\text{average}}$ has two components. One is the systematic difference between the calculated image and the scanned image (i.e., the high‐contrast phantom image), and the other is the image noise level of the scanned image as the calculated image was obtained with the noise‐free simulation. This systematic difference is dependent on the MTF accuracy (i.e., the ROI size in the MTF measurements with wire/bead methods). However, the scanned image noise is independent of the MTF accuracy. In the MTF verification, the calculated images were changed using the MTFs, while the scanned image was constant, indicating that the scanned image noise for the ${\text{SD}}_{\text{average}}$ was constant. Thus, the ${\text{SD}}_{\text{average}}$ changed because of the systematic difference, with inclusion of the constant noise on the scanned image. To show this image noise level, we defined the ‘${\text{SD}}_{\text{FC}52}$’ as the SD obtained in the ROI on the scanned image (see Fig. 3). The ${\text{SD}}_{\text{FC}10},{\text{SD}}_{\text{FC}50}$, and ${\text{SD}}_{\text{FC}52}$ were obtained on each image reconstructed with the kernels of FC10, FC50, and FC52, respectively (described next, in Section C).

### C. Equipment and imaging parameters

A 4 multidetector‐row CT (Asteion4; Toshiba Medical Systems, Tokyo, Japan) was used. In the MTF measurements, we scanned the wire and bead phantoms using 120 kV, 200 mA, and 0.75 sec/rot; the image reconstruction parameters were 0.5 mm slice thickness, 50 mm field of view (FOV), and 512 matrix. In the MTF verification, we scanned the high‐contrast CT test phantom using 120 kV, 200 mA, and 0.75 sec/rot; the image reconstruction parameters were 1.0 mm slice thickness, 100 mm FOV, and 512 matrix. We chose three types of reconstruction kernels as ‘smooth’ (FC10, for standard abdominal imaging), ‘standard’ (FC50, for standard lung imaging), and ‘edge‐enhancement’ (FC52, for high‐resolution lung imaging). All calculations were performed using the technical computing software MATLAB (The MathWorks Inc., Natick, MA).

## III. RESULTS

### A. MTF measurements by wire and bead methods

**Figure 5:**
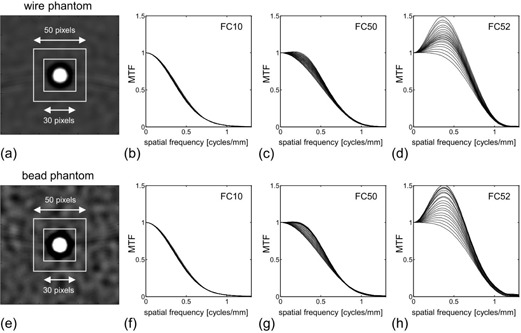
MTFs obtained from wire and bead phantoms. Wire phantom image (a) reconstructed with the kernel FC52. Two ROIs (30 and 50 pixels) were placed on the image. Twenty‐one MTF results (b) were obtained by changing the ROI size from 30 to 50 pixels on the wire image reconstructed with the kernel FC10. Twenty‐one MTF results (c) for the kernel FC50. Twenty‐one MTF results (d) for the kernel FC52. Results (e)–(h) for the bead method are as for (a)–(d).

We obtained the MTFs from the wire and bead phantom images (Fig. 5). Two ROIs with 30 and 50 pixel sizes were indicated in the wire image (Fig. 5(a)). By changing the ROI size from 30 to 50 pixels (increment of 1 pixel), 21 results of MTFs for FC10 were obtained (Fig. 5(b)). In the same way, by changing the ROI size on the images for the kernels of FC50 and FC52, the results of MTFs were also obtained for FC50 (Fig. 5(c)) and FC52 (Fig. 5(d)). While the MTFs for FC10 and FC50 were largely unaffected by the ROI size, the MTFs for FC52 showed a large fluctuation. The results for the bead method are shown in Figs. 5(e)–5(h), corresponding to those for the wire method in Figs. 5(a)–5(d); similar results were obtained between these two methods. In both methods, the MTFs for the edge‐enhancement‐type kernel (FC52) were strongly dependent on the ROI size. The MTF values tended to increase as the ROI size was increased. However, when the ROI size was increased more than 45 pixels, MTF values only for FC52 obtained by the bead method (Fig. 5(h)) showed a random variation without the increasing trend.

### B. MTF determination using the verification method

**Figure 6:**
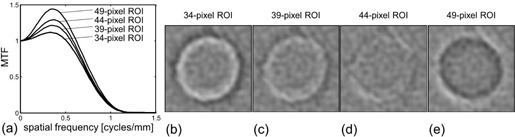
Results of verification for four MTFs. Four results of MTF (a) obtained by the wire method with the ROI sizes of 34, 39, 44, and 49 pixels for the reconstruction kernel of FC52. Images (b) obtained by subtracting the scanned images from the calculated images in the verification for the MTF obtained with a 34 pixel ROI. One subtraction image for 10 mm diameter cylinder is shown (those for 7, 5, 3, and 2 mm diameter cylinder are not shown). Result of the verification for the MTF (c) obtained with a 39 pixel ROI is as for (b). Result of the verification for the MTF (d) obtained with a 44 pixel ROI is as for (b). Result of the verification for the MTF (e) obtained with a 49 pixel ROI is as for (b). Window settings were C/W=0/300HU in (b)–(e).

The results of verification for four MTFs are illustrated in Fig. 6. The MTFs were obtained by the wire method with ROI sizes of 34, 39, 44, and 49 pixels for the reconstruction kernel of FC52. These MTFs shown in Fig. 6(a) were validated by the verification method in which the subtraction images (corresponding to Fig. 4(c)), indicating the accuracy of MTF, were obtained. The subtraction images (for 10 mm diameter cylinder) for the MTF obtained with the 34‐, 39‐, 44‐, and 49‐pixel ROIs are shown in Figs. 6(b)–6(e), respectively. The residual errors on the subtraction images were found around the cylinder edge and were changed by the ROI size. The subtraction images for the MTF obtained with the ROI size of 44 pixels showed fewer residual errors than those for the MTFs with the other ROI sizes. The values of ${\text{SD}}_{\text{average}}$ were 33.9, 20.5, 15.6, and 31.5 HU for the 34‐, 39‐, 44‐, and 49‐pixel ROIs, respectively. The MTF with the 44‐pixel ROI was confirmed to be more accurate than those with the other ROI sizes.

**Figure 7:**
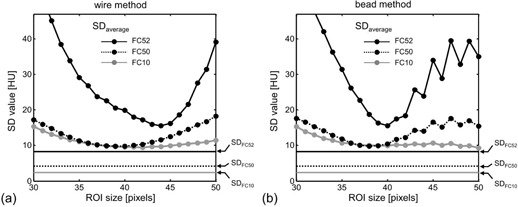
Values of ${\text{SD}}_{\text{average}}$ for the MTFs obtained by changing the ROI size in the wire method (a) and the bead method (b) for the reconstruction kernels of FC10, FC50, and FC52. The noise in the high‐contrast phantom image for each reconstruction kernel was evaluated by the standard deviation of ${\text{SD}}_{\text{FC}10},{\text{SD}}_{\text{FC}50}$, and ${\text{SD}}_{\text{FC}52}$ (see Fig. 3).

All MTFs obtained by changing the ROI sizes in the wire/bead methods were validated by the verification method, in which the values of ${\text{SD}}_{\text{average}}$ were obtained (Fig. 7). For the wire method (Fig. 7(a)), the ${\text{SD}}_{\text{average}}$ showed the smallest values at an ROI size of 42 pixels for FC10, 39 pixels for FC50, and 44 pixels for FC52. For the bead method (Fig. 7(b)), the ${\text{SD}}_{\text{average}}$ showed the smallest values at an ROI size of 50 pixels for FC10, 38 pixels for FC50, and 40 pixels for FC52. These ROI sizes were optimum for obtaining accurate MTFs and were different depending on the reconstruction kernels and the MTF measurement methods (wire vs. bead). The noise on the high‐contrast phantom image used in the verification method was evaluated by the ${\text{SD}}_{\text{FC}10},{\text{SD}}_{\text{FC}50}$, and ${\text{SD}}_{\text{FC}52}$ (Fig. 7). The values of ${\text{SD}}_{\text{FC}10},{\text{SD}}_{\text{FC}50}$, and ${\text{SD}}_{\text{FC}52}$ were 2.4, 4.2, and 8.2 HU, respectively.

**Figure 8:**
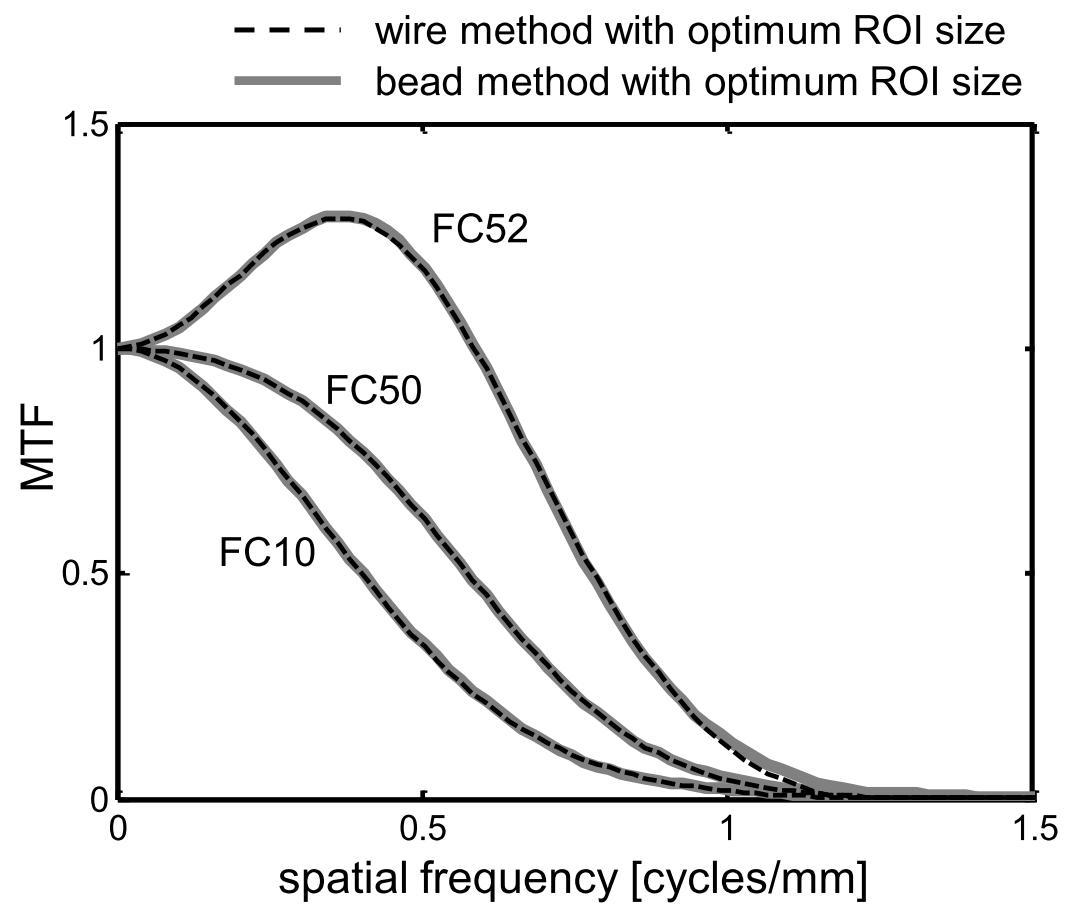
Comparison of MTFs obtained by the wire and bead methods with the optimum ROI sizes for the reconstruction kernels of FC10, FC50, and FC52.

With the optimum ROI sizes determined by the verification method (Fig. 7), the MTFs were obtained by the wire and bead methods for three reconstruction kernels (Fig. 8). The MTFs obtained by the wire method agreed well with those obtained by the bead method for all kernels. Accurate and precise MTF determinations were demonstrated.

## IV. DISCUSSION

MTF measurements are often performed using the wire and bead methods, which involve image data processing by integrating an ROI. However, the ROI size can affect the resultant MTF, especially for the MTF measurement of edge‐enhancement‐type kernels such as FC52, making it difficult to obtain accurate measurements. As the ROI size is generally decided empirically, the MTFs determined using the wire and bead methods are not always accurate. By incorporating the verification method into the wire and bead methods, the optimum ROI size can be determined (see Fig. 7), allowing accurate and precise determination of MTF.

We found that the MTFs measured by the wire and bead methods were dependent on ROI size (Fig. 5); this effect was particularly obvious for special edge‐enhancement kernels such as FC52. To allow accurate MTF calculation, the ROI has to include a large enough area to avoid the truncation of the under‐ or overshooting in PSF. However, if the ROI includes too large an area, extra noise and artifacts may also be included, potentially distorting the results. Therefore, the appropriate ROI size needs to be selected. In the images of the object (wire/bead) response used in the present study (Figs. 5(a) and 5(e)), the undershoot response (indicated as a dark region surrounding the object) was almost included by the 30‐pixel ROI; we regarded this ROI size as a minimum visually. The area outside the 30‐pixel ROI may include a slight component of the object response, while the area outside the 50‐pixel ROI would include almost no component of object response. Therefore, we investigated the effect of ROI sizes from 30 to 50 pixels. In MTF measurement for reconstruction kernel of FC52, the optimum ROI sizes were 44 pixels and 40 pixels for the wire and the bead methods, respectively (Fig. 7). When the ROI size was increased more than the optimum size, the measurement error of MTF (SD value in Fig. 7) was increased, indicating that a larger ROI included extra noise and artifacts. Thus, the obtained optimum ROI sizes were large enough to include the entire object response without including extraneous noise and artifact, and the validity of the ROI size setting was in the range of 30 to 50 pixels.

The ${\text{SD}}_{\text{average}}$ curve for FC52 in the bead method (Fig. 7(b)) was observed to oscillate as the ROI size was increased to more than approximately 42 pixels. When the ROI size was increased more than the optimum size, extra noise and artifacts are included, as discussed above. As a 1 pixel increase of the ROI size was performed by enlarging the ROI alternately on left and upper sides and on right and lower sides, the effects of extra noise and artifacts change alternately.

Since the noise in the bead image for FC52 was large (Fig. 5(e)), the ${\text{SD}}_{\text{average}}$ curve for FC52 in the bead method showed an obvious oscillation. Further, this phenomenon suggested that the ROI size of more than approximately 42 pixels included extra noise and artifacts (i.e., the ROI size was too large).

Detailed methodologies for the wire and bead methods have not been investigated for currently available MDCT. In a typical approach for MTF determination,^7^, ^8^, ^9^, ^13^, ^14^, ^15^, ^16^, ^17^, ^18^, ^19^ the LSF is first obtained by processing the wire/bead image data. In the present study, to determine the LSF, the square‐shaped ROI was integrated and the obtained data were then offset‐corrected and normalized (Fig. 2). Other methods can be used during this procedure, such as rectangular‐shaped ROIs^13^, ^14^, ^17^ and the inclusion of further processing (e.g., noise/artifact reduction).^(12)^ The results of the MTF fluctuations shown in Fig. 5 can change depending on the processing methods used. Further, in such cases, the verification method^(26)^ can be applied to optimize the various processing parameters using the same approach as in our study. In addition, the verification method can be incorporated into any MTF measurement method^10^, ^11^, ^12^, ^13^, ^14^, ^15^, ^16^, ^17^, ^18^, ^19^, ^20^, ^21^, ^22^, ^25^ for the validation of measured MTFs and/or for the optimization of processing parameters.

In the wire and bead methods, the ROI setting was essential for obtaining the LSF, indicating that ROI size dependency is a potential problem in these techniques regardless of the type of CT scanners. Even when we used the most modern machines such as a 64‐slice or higher MDCT, the problem of the ROI size dependency remained, and the verification method was applicable for accurate MTF measurements, as demonstrated by this study using a 4‐slice CT.

## V. CONCLUSIONS

We demonstrated that MTFs measured by the wire and bead methods were dependent on the size of the ROI used for obtaining the LSF, particularly in MTF measurement for image reconstruction kernel of edge‐enhancement type. By incorporating the verification method into the wire and bead methods, the ROI sizes could be optimized, resulting in accurate and precise determination of MTFs and effective evaluation of image quality. We recommend the use of this verification method when performing the wire and bead methods for some special kernels such as edge‐enhancement.

## ACKNOWLEDGMENTS

This study was supported in part by a Grant‐in‐Aid for Cancer Research (19–25) from the Ministry of Health, Labour and Welfare, Japan, and by a Grant‐in‐Aid for Scientific Research (C) (23602005) and a Grant‐in‐Aid for Scientific Research on Priority Areas (15070205) from the Ministry of Education, Culture, Sports, Science and Technology of Japan. This study was also supported jointly by Niigata University and Fujitsu Limited.

## Supporting information

Supplementary Material
